# Relationship between Dietary Behaviors and Physical Activity and the Components of Metabolic Syndrome: A Case-Control Study

**DOI:** 10.3390/ijerph19116562

**Published:** 2022-05-27

**Authors:** Małgorzata Godala, Michalina Krzyżak, Dominik Maślach, Ewelina Gaszyńska

**Affiliations:** 1 Department of Nutrition and Epidemiology, Medical University of Lodz, 90-419 Lodz, Poland; ewelina.gaszynska@umed.lodz.pl; 2 Department of Hygiene, Epidemiology and Ergonomics, Medical University of Bialystok, 15-089 Bialystok, Poland; michalina.krzyzak@umb.edu.pl; 3 Department of Public Health, Medical University of Bialystok, 15-089 Bialystok, Poland; dominikm@umb.edu.pl

**Keywords:** diet, physical activity, nutrition knowledge, metabolic syndrome, lifestyle, dietary behaviors

## Abstract

Poor diet and low physical activity play an important role in the etiopathogenesis of metabolic syndrome. The aim of this study was to analyze the association between nutrient intake, groups of food products and physical exercise undertaken and the components of metabolic syndrome (MS). The study included 330 patients with MS, and the control group comprised of 270 subjects without MS. The food intake was assessed using 24-h dietary recall, and a 13-item Food Frequency Questionnaire. To assess nutrition knowledge, a Beliefs and Eating Habits Questionnaire was used. The level of physical activity was assessed using the International Physical Activity Questionnaire. Three patterns of behavior were identified: Prudent-Active, Western-Sedentary, and NotPrudent-notWestern-lowActive. In the Prudent-Active group, as compared to the NotPrudent-notWestern-lowActive subjects, the risk of central obesity, hypertension, hypertriglyceridemia, low HDL cholesterol and hyperglycemia occurrence was lower. There was also a lower proportion of patients with MS. As compared to the NotPrudent-notWestern-lowActive subjects, in the Prudent-Active group there was more than a two times higher chance of subjects with a high level of nutrition knowledge. Western diets have been proven to exert a detrimental effect on the components of MS. When designing intervention programs, education of patients with MS on dietary habits and physical activity should be considered.

## 1. Introduction

Metabolic syndrome (MS) is a group of conditions including central obesity, hypertension, and lipid and carbohydrate metabolism disorders considered a risk factor for cardiovascular diseases, the leading cause of death both in Europe and worldwide [1,2,3]. Studies conducted in recent years indicate an increasing percentage of individuals meeting the MS criteria. The prevalence of the syndrome varies from a few to several dozen percent, depending on the country, age of the subjects and the applied definition [4,5]. Undoubtedly, central obesity, which is the main criterion for diagnosing MS, is the major risk factor for insulin resistance and inflammation frequently occurring in patients with cardiovascular diseases [6].

As confirmed by many studies, poor diet and low physical activity play an important role in the etiopathogenesis of metabolic syndrome [7,8,9]. The influence of selected dietary models is also often discussed. Among them, the Mediterranean diet and DASH (Dietary Approaches to Stop Hypertension) are those most commonly recommended for patients with MS and cardiovascular diseases [10,11]. Studies have shown positive effects of low-fat diets as well as diets rich in fruits, vegetables, fish and high-fiber products on components of MS and inflammatory parameters. In contrast, eating habits based on animal products, saturated fatty acids, and refined sugars showed a negative association with both the components of MS and its incidence [12,13,14].

There are results of studies evaluating the influence of selected nutrients, food groups or even specific dietary models on the occurrence of lifestyle diseases such as obesity, hypertension, type 2 diabetes and cardiovascular diseases [15,16,17]. However, the issue still needs to be discussed due to the rapidly changing food market, and newly emerging dietary recommendations often challenging generally accepted patterns, such as high-protein, high-fat and low-carbohydrate diets [16,18]. In addition, the lifestyle of patients, especially in the context of their physical activity, is also important in the etiopathogenesis of metabolic diseases [11,14,19,20].

The aim of this study was to analyze the association between nutrient intake, groups of food products and physical exercise undertaken, and the components of MS.

## 2. Materials and Methods

### 2.1. Study Population

The study included 330 patients with MS, 171 men and 159 women, aged 30–67 years (mean 55.2 ± 7.9 years). The control group comprised of 270 subjects, 143 men and 127 women, aged 41–63 years (mean 56.2 ± 4.9 years), clinically healthy, without MS. All participants were recruited from the Department of Internal Medicine and Nephrodiabetology and Department of Nutrition and Epidemiology, Medical University of Lodz.

### 2.2. Metabolic Syndrome (Definition)

The MS diagnosis was based on IDF (International Diabetes Federation) criteria, stating the type of central obesity (waist circumference in women ≥ 80 cm, in men ≥ 94 cm) and two of the following risk factors: triglycerides ≥ 1.7 mmol/L or treatment of hyper-tryacylglycerolemia, low HDL cholesterol (in women < 1.3 mmol/L, in men < 1.0 mmo/L) or treatment of the disorder, fasting glucose level ≥ 6.1 mmol/L or treated type 2 diabetes, blood pressure ≥ 130/85 mmHg or treatment of hypertension [1].

### 2.3. Biochemical Analyses

The mean of three blood pressure (systolic and diastolic) readings was measured on three different days using a mercury sphygmomanometer by professional medical staff. The participants rested before the test, sitting down and relaxing on a chair, and waiting about three minutes before taking a measurement. Fasting blood samples were drawn from the basilic vein of the subjects for laboratory examination. Fasting blood glucose was determined with a reaction between glucose and ATP catalyzed by hexokinase; TG (triglycerides) concentration was enzymatically measured with coupled reactions in which TG was hydrolyzed to produce glycerol; TC (total cholesterol) was measured with reactions using cholesteryl ester hydrolase, cholesterol oxidase, and peroxidase; HDL (high density lipoprotein) was measured using a heparin-manganese precipitation method; LDL (low density lipoprotein) was calculated using the Friedewald rule.

### 2.4. Nutritional Evaluation

The food intake was assessed using a questionnaire concerning ingestion within the last 72 h prior to the examination, in accordance with the guidelines of the National Food and Nutrition Institute in Warsaw [21]. About 1800 twenty 24 dietary recalls (three 24-h dietary recalls for each individual) were obtained from all participants by the interviewer, and means of consumption for each nutrient were calculated. The “Album of photographs of food products and dishes” of the National Food and Nutrition Institute in Warsaw was used to determine normal size of the consumed portions [22].

Additionally, dietary data were obtained by a 13-item Food Frequency Questionnaire (FFQ) using the previous week/month as a reference period. Participants were asked to assess frequency of consumption as servings per day or per week for each food group.

### 2.5. Nutrition Knowledge

To assess nutrition knowledge of participants, the Beliefs and Eating Habits Questionnaire (part C) created by the Behavioral Conditions of Nutrition Team, Committee of Human Nutrition Science, Polish Academy of Science was used [23]. The questionnaire consists of 25 questions, each correct answer was scored with 1 point, and incorrect or “I don’t know” answers were scored with 0 point. Three levels of nutrition knowledge categories were derived: insufficient (0–8 points), sufficient (9–16 points) and high (17–25 points).

### 2.6. Physical Activity

In all the studied subjects, the level of physical activity was assessed using the International Physical Activity Questionnaire Long Form (IPAQ), consisting of a 27-item self-reported measure of physical activity [24]. The authors estimated the total physical activity expressed in MET-min/week (Metabolic Equivalent of Work), where one MET represents resting energy expenditure assuming oxygen consumption of 3.5 mL/min/kg body weight.

There are three types of effort: light (walking), moderate (with slightly increased respiratory rate and slightly accelerated heart rate, such as carrying light objects, cycling at a normal speed, brisk walking) and intensive (with highly increased respiratory rate and accelerated heart rate, such as aerobics, fast cycling). The time spent on each type of activity in the last week was recorded and only efforts lasting at least 10 min were taken into account.

The questionnaire consists of 27 questions divided into five groups, each of which contains detailed questions on intensive, moderate and walking activities undertaken by the subjects in the last week and related to their work, active mobility, housework, leisure and sport. Moreover, time spent inactively during the week and at weekends was also analyzed in the questionnaire.

The results were classified according to the following criteria:

1. insufficient physical activity (less than 600 MET-min/week);
2. sufficient physical activity (between 600 and 1500 MET-min/week);
3. increased physical activity (1500–3000 MET-min/week, but less than 3 days per week of intense exercise);
4. high physical activity (above 1500 MET-min/week but at least 3 days per week of intense exercise, or at least 3000 MET-min/week).

The respondents completed the questionnaire with the help of a questioner. The questionnaire was thoroughly discussed prior to the commencement of the study, and particular attention was paid to explanation of the terminology used in the questionnaire and the interpretation of the intensity of physical effort. For each category, the respondents separately identified the number of days and time spent on intensive and moderate activity as well as walking.

### 2.7. Anthropometry

All the subjects had their waist circumference, hip circumference, body height and weight measured. Waist circumference was measured at the level of umbilicus with an accuracy of 0.5 cm. The subject stood erect with relaxed abdominal muscles, arms at the side, and feet together. The measurement was taken at the end of a normal expiration. Hip circumference was measured at the point of greatest circumference around hips and buttocks with an accuracy of 0.5 cm. Both measurements were taken with a flexible, non-stretchable tape in close contact with the skin but without indenting the soft tissue. To measure height, the subject stood erect and barefoot on a stadiometer with a movable head piece. The head piece was aligned with the skull vault and height was recorded at an accuracy of 0.5 cm. To measure weight, a regularly calibrated electronic scale was used. The subjects were weighed in light clothes, without shoes. Weight was recorded at an accuracy of 100 g. WHR was determined by dividing the waist circumference by hip circumference, and BMI (body mass index) was determined by dividing body weight expressed in kilograms by height in square meters.

The body composition of the subjects was assessed with electrical bio-impedance analysis (BIA), using a Bodystat 1500 MDD apparatus. The measurement was taken at least 4 h after the last meal, with a four-electrode system; the patients were examined in a supine position, without contact with the metal object. Fat body mass and fat-free body mass were measured.

### 2.8. Statistical Analysis

Statistical analysis was performed using the Statistica v.13 program. Descriptive statistics with determination of the mean and standard deviation or median and interquartile range were made. Categorical variables were presented in the form of number and percentages. The analysis of compatibility of the variable distribution with the normal distribution was performed with the application of the Shapiro-Wilk test. When the analyzed variables appeared to be incompatible with the normal distribution, the authors used the Mann-Whitney test to compare the study and control groups. Cluster analysis was used to derive dietary-lifestyle patterns (DLP). Variables used in the analysis were 13 food groups (in times per day) and the level of physical activity (in scores) as components of DLPs. To classify the subjects, K-means clustering algorithm was used based on the Euclidean distances. The authenticity of cluster identification was checked by comparing the components of DPLs between clusters with a one-way analysis of variance. The odds ratios (OR) with 95% confidence intervals (95% CI) were measured and crude models were created. Wald statistics were used to assess the significance of ORs. *p* < 0.05 was considered as significant for all used tests.

## 3. Results

Higher prevalence of obesity, hypertension and hyperlipidemia was observed in the group with MS. Those subjects also presented higher mean levels of glucose, total cholesterol and LDL cholesterol as well as lower levels of HDL cholesterol as compared to the control group (Table 1). Patients with MS showed higher total protein intake than the healthy subjects and lower physical activity and nutritional knowledge than the control group (Table 2).

Based on the frequency of food intake and physical activity of the subjects, three patterns of behavior were identified, i.e., Prudent-Active, Western-Sedentary, and NotPrudent-notWestern-lowActive. The subjects in the Prudent-Active group were characterized by a high intake of fruits, vegetables, whole grains, dairy products and fish, and moderate to high levels of physical activity. The Western-Sedentary group was characterized by a high intake of fast-food products, white bread, red meat, sweets, sweetened beverages, and low to moderate levels of physical activity. The NotPrudent-notWestern-lowActive group was characterized by a low intake of vegetables, fruits, fish, fast foods, sweetened beverages, sweets, and moderate or adequate levels of physical activity.

As compared to the other DLPs, among the subjects assigned to the Prudent-Active group, there was a lower mean age, a higher proportion of women and a lower proportion of patients with MS. Additionally, the subjects included in the Prudent-Active DLP showed lower levels of glucose, total cholesterol, LDL cholesterol and TG, but higher mean levels of HDL cholesterol. Hypertension, hypercholesterolemia and central obesity were less common in this group (Table 3). There were no significant differences observed in energy or nutrient intake between the DLPs except for protein and fats whose lower intake was characteristic for the Prudent-Active group. The Prudent-Active subjects also demonstrated a broader nutritional knowledge than the other DLPs.

As compared to the NotPrudent-notWestern-lowActive subjects, in the Prudent-Active group there was a higher percentage of women, individuals with sufficient nutrition knowledge, and a more than a two times higher chance of subjects with a high level of nutrition knowledge (Table 4). In the Western-Sedentary group, as compared to the NotPrudent-notWestern-lowActive group, there was a 17% lower percentage of women, 24% lower percentage of those with sufficient nutrition knowledge and 57% of those with high nutrition knowledge. Among the Western-Sedentary subjects, as compared to the Prudent-Active group, there was a 53% lower percentage of women, 58% lower percentage of individuals with sufficient nutrition knowledge and 77% lower percentage of those with high level of nutrition knowledge. The highest number of subjects with MS was recorded in the Western-Sedentary group as compared to the Prudent-Active group and the NotPrudent-notWestern-lowActive group.

In the Prudent-Active group, as compared to the NotPrudent-notWestern-lowActive subjects, the risk of central obesity occurrence was 43% lower. In the case of hypertension, the risk was lower by 48%, hypertriglyceridemia by 41%, low HDL cholesterol by 29% and hyperglycemia by 38% (Table 5). There were no significant relations between the prevalence of central obesity, hypertension, hypertriglyceridemia or hyperglycemia in the Western-Sedentary group as compared to the NotPrudent-notWestern-lowActive group. In contrast, the risk of low HDL cholesterol occurrence was found to be 17% higher. The Western-Sedentary group showed a more than twofold higher risk of central obesity, hypertriglyceridemia and low HDL cholesterol than the Prudent-Active group. Additionally, there was a 76% higher risk of hypertension and a 34% higher risk of hyperglycemia.

## 4. Discussion

The study proved a relationship between dietary behavior and physical activity with metabolic syndrome parameters. Of the three behavioral patterns identified, the strongest associations with the components of metabolic syndrome and the highest risk of MS development were demonstrated in the Western-Sedentary group. The prevalence of MS was estimated to be 78.8% in the Western-Sedentary group, 52.9% in the NotPrudent-notWestern-lowActive group, and 9% in the Prudent-Active group. Significant differences were also identified in the frequency and intensity of abnormalities related to MS, with unfavorable parameters found significantly more often in the Western-Sedentary group than in the other cohorts. There were also significant differences in the frequency of consumption of food groups among the subjects with MS as compared to the control cohort, with a higher frequency of consumption of health-promoting products in the control group and a higher frequency of consumption of pro-inflammatory products among patients with MS.

The relationship between broadly-defined lifestyle and metabolic disorders has been known for a long time. However, it still needs to be discussed, and the influence of individual lifestyle elements, including diet, on the occurrence of inflammatory and metabolic changes has to be assessed. To our knowledge, this is the first Polish study to evaluate the relationship between diet and physical activity and the components of metabolic syndrome. In many aspects, the results of our study confirmed the data obtained by other authors. Studies conducted in Europe and worldwide have demonstrated the impact of high intake of meat, especially red meat, refined sugars and fast-food products with a high frequency of MS components [7,25,26,27]. An inverse relationship has also been proven between the incidence of MS components and high consumption of products such as vegetables and fruits, dairy and cereal products [7,9,14,28]. Our study demonstrated a more than twofold higher risk of MS in the Western-Sedentary group (as compared to the Prudent-Active group), characterized by low to moderate levels of physical activity and high consumption of fast-food products, white bread, red meat, sweets and sweetened beverages. In contrast, there are also reports not confirming any relationship between MS components and poor eating habits, although these represent a large minority [8,29].

Our study showed significant differences in the intake of specific food groups between patients with MS and the controls, with higher intake of non-recommended foods observed in the group with MS. In contrast, there were no significant differences in the intake of energy and most nutrients, with the exception of total protein intake, which was found to be higher in the diet of the patients with MS. The data indicate an important role of developing proper eating habits in terms of specific groups of food products chosen, regardless of the quantitative assessment of the intake of particular nutrients or energy. Our results suggest that it may be the qualitative assessment of diet that is more important than analysis of its nutritional value. This is of particular importance in the case of fats and the content of individual fatty acids. In our study, we found a significantly higher intake of fish and vegetable oils in the group of healthy subjects than in those with MS, as well as differences in the intake of monounsaturated and polyunsaturated fatty acids intake, which was significantly higher in the group of healthy subjects. Numerous studies have shown the positive effect of a high intake of fish and unsaturated fatty acids on the lipid profile, CRP levels, incidence of metabolic syndrome and cardiovascular events [2,3,12,18,30]. The results of our study lead to similar conclusions.

Among the components of metabolic syndrome, the strongest relationships with the selected behavioral patterns were found for HDL cholesterol, triglycerides and waist circumference. The risk of low HDL cholesterol, high triglycerides and waist circumference indicating central obesity were more than twice as high in the Western-Sedentary group as compared to the Prudent-Active group. In contrast, the Prudent-Active group was nearly twice as likely to have all the components of MS as compared with the NotPrudent-notWestern-lowActive group. Similar data were obtained in other studies [25,26]. The relationship between diet and intake of selected nutrients with and all components of MS has long been recognized. Studies have demonstrated the effect of Western sedentary lifestyle patterns and a diet rich in animal products on increased risk of MS, cardiovascular and gastrointestinal diseases [5,31]. Our study also proves such an association, further indicating the influence of bad eating habits on TG and LDL levels, which has not been confirmed by other studies [8,29].

In the literature, there are discussions about the impact diet has, not only on the therapeutic management of metabolic disorders, but also on the prevention of these diseases [31,32,33]. In terms of efforts aimed at broadening knowledge of risk factors for metabolic and cardiovascular diseases, patient education seems to be a key element for increasing health awareness. Our study demonstrates lower nutritional knowledge of subjects with MS as compared to healthy individuals, and a higher percentage of the subjects with sufficient and high nutritional knowledge in the Prudent-Active group as compared to the NotPrudent-notWestern-lowActive group. At the same time, the number of individuals with high nutritional knowledge in the Western-Sedentary group was 77% lower than that in the Prudent-Active group. Studies verifying the nutritional awareness and behaviors of patients with metabolic disorders stress not only the role of knowledge of risk factors for these diseases, but also the ability to counteract them in practice. Studies have shown a large variation in the knowledge of risk factors among patients with MS, although they declare a strong need to be aware of these factors [32,33]. Active lifestyle and rational diet are key elements of the therapeutic management of patients with MS, so when designing intervention programs a great emphasis should be put on increasing nutritional knowledge in this group of patients.

The conducted study has some limitations. Due to the low level of diversity of the group, it was impossible to assess the influence of economic status on the analyzed parameters. When estimating the frequency of consumption of food groups based on a food frequency questionnaire (FFQ), we used standard portion sizes. These correspond to approximate values of consumption and are automatically subject to certain inaccuracies, therefore they do not take individual differences into account.

## 5. Conclusions

Western diets have been proven to exert a detrimental effect on the components of metabolic syndrome and to increase the risk of disease occurrence. A relationship has also been shown between low intake of foods such as vegetables, fruits, whole grain cereal products, combined with high intake of fast food, white bread, red meat, sweets, sweetened beverages, and the presence of metabolic syndrome. When designing intervention programs, education of patients with MS on dietary habits and physical activity should be considered.

## Figures and Tables

**Table 1:** General characteristics of study population.

Characteristics	MS (n = 330) Mean ± SD/n (%)	Control (n = 270) Mean ± SD/n (%)	*p*-Value
Age (years)	55.2 ± 7.9	56.2 ± 4.9	0.312
Sex (% women)	159 (48.2)	127 (47.0)	0.357
Hypertension	215 (65.2)	51 (18.8)	<0.001
Use of antihypertensives	118 (35.7)	14 (5.2)	<0.001
Hyperlipidemia	220 (66.7)	76 (28.1)	<0.001
Use of hypolipidemics	42 (12.7)	11 (4.1)	<0.001
Systolic Blood Pressure (mmHg)	133.5 ± 5.9	112.5 ± 7.1	<0.001
Diastolic Blood Pressure (mmHg)	89.3 ± 6.2	76.8 ± 5.4	<0.001
Fasting Glucose (nmol/L)	5.1 ± 0.6	4.6 ± 0.5	<0.001
Triglycerides (nmol/L)	1.5 ± 0.8	0.9 ± 0.6	<0.001
Total Cholesterol (nmol/L)	5.6 ± 1.2	4.8 ± 1.1	<0.001
HDL Cholesterol (nmol/L)	1.2 ± 0.7	1.6 ± 0.5	<0.001
LDL Cholesterol (nmol/L)	3.2 ± 1.5	2.8 ± 1.4	<0.001
BMI (kg/m^2^)	29.3 ± 4.7	23.8 ± 5.8	<0.001
BMI categories			
Underweight	0 (0)	11 (4.1)	<0.001
Normal weight	33 (10)	181 (67.1)	
Overweight	176 (53.3)	62 (22.9)	
Obesity	121 (36.7)	16 (5.9)	
Waist circumference (cm)	104.6 ± 12.9	89.7 ± 11.6	<0.001
WHR	0.95 ± 0.13	0.81 ± 0.11	<0.001
Central obesity (%)	330 (100)	152 (56.3)	<0.001
Body composition			
Body mass (kg)	82.5 ± 7.8	76.5 ± 8.7	<0.0001
Fat free mass (kg)	42.9 ± 7.2	48.7 ± 5.9	<0.0001
Fat mass (kg)	37.4 ± 9.1	27.4 ± 8.3	<0.0001
Fat mass (%)	39.5 ± 7.9	34.7 ± 4.4	<0.0001

HDL—High Density Lipoprotein, LDL—Low Density Lipoprotein, WHR—waist-hip ratio, BMI—body mass index.

**Table 2:** Physical activity and dietary characteristics of study population.

Characteristics	MS (n = 330) Mean ± SD/n (%)	Control (n = 270) Mean ± SD/n (%)	*p*-Value
Physical activity			
Insufficient	180 (54.5)	28 (10.4)	<0.0001
Sufficient	90 (27.3)	140 (51.8)	
Increased	47 (14.2)	91 (33.7)	
High	13 (3.9)	11 (4.1)	
Dietary intake			
Total energy intake (kcal/day)	2112.3 ± 553.1	1920.2 ± 418.4	0.0673
Proteins (g/day)	107.1 ± 38.8	96.6 ± 28.3	0.0351
Proteins (% total energy intake)	19.7 ± 4.5	20.4 ± 3.5	0.0143
Carbohydrates (g/day)	278.1 ± 98.2	277.3 ± 79.2	0.1284
Carbohydrates (% total energy intake)	52.3 ± 6.8	47.1 ± 8.1	0.0565
Fats (g/day)	84.7 ± 53.9	76.9 ± 21.1	0.0612
Fats (% total energy intake)	36.6 ± 7.1	33.4 ± 7.2	0.0704
MUFA	29.9 ± 24.1	33.2 ± 17.7	0.0347
PUFA	9.9 ± 8.1	12.9 ± 6.1	0.0295
SFA	38.4 ± 22.7	31.1 ± 16.9	0.0127
Cholesterol (mg)	275.9 ± 16.3	221.8 ± 24.7	0.1053
Frequency of consumption (times per day, median and interquartile range)			
Vegetables	1.1 (0.5–1.5)	1.7 (0.7–3.5)	<0.001
Fruits	1.5 (0.5–2.1)	2.7 (0.7–2.9)	<0.001
Whole grain products	1.2 (0.3–2.2)	3.2 (1.0–4.5)	<0.001
White meat products	3.5 (1.0–4.1)	2.1 (1.0–4.1)	<0.001
Red meat	1.6 (0.3–2.5)	1.1 (0.5–2.4)	<0.001
Poultry	1.5 (0.9–2.5)	1.7 (0.5–2.6)	0.0765
Fish	0.3 (0.1–0.9)	0.5 (0.5–1.3)	0.0412
Dairy products	1.0 (0.5–1.9)	1.9 (0.5–2.7)	<0.001
Butter	2.1 (0.5–3.0)	2.2 (0.5–3.1)	0.0624
Plant oils	0.7 (0.0-1.7)	1.5 (0.5–2.2)	<0.001
Fast foods	1.5 (0.5–3.1)	0.5 (0.5–1.9)	<0.001
Sweetened beverages	1.8 (0.5–3.0)	0.5 (0.5–1.5)	<0.001
Sweets	2.1 (0.5–4.1)	0.7 (0.5–2.0)	<0.001
Nutrition knowledge			
Insufficient (%)	162 (49.1)	37 (13.7)	<0.001
Sufficient (%)	141 (42.7)	152 (56.3)	
High (%)	27 (8.2)	81 (30)	

MUFA—monounsaturated fatty acids, PUFA—polyunsaturated fatty acids, SFA—saturated fatty acids.

**Table 3:** Study population according to dietary-lifestyle patterns.

Variables	Prudent-Active (n = 122) Mean ± SD/n (%)	NotPrudent-NotWestern-LowActive (n = 223) Mean ± SD/n (%)	Western-Sedentary (n = 255) Mean ± SD/n (%)	*p*-Value
Age (years)	49.6 ± 2.3	51.3 ± 5.1	56.0 ± 6.3	<0.001
Sex (% women)	70 (57.4)	122 (54.7)	94 (36.9)	<0.001
MS (%)	11 (9.0)	118 (52.9)	201 (78.8)	<0.001
Hypertension	22 (18.0)	89 (39.9)	155 (60.8)	<0.001
Hyperlipidemia	18 (14.8)	110 (49.3)	168 (65.9)	<0.001
Fasting Glucose (nmol/L)	4.6± 0.4	4.9 ± 0.7	5.1 ± 0.6	<0.001
Triglycerides (nmol/L)	0.9 ± 0.5	1.3 ± 0.3	1.4 ± 0.9	<0.001
Total Cholesterol (nmol/L)	4.6 ± 0.9	5.2 ± 0.8	5.7 ± 0.5	<0.001
HDL Cholesterol (nmol/L)	1.5 ± 0.7	1.2 ± 0.5	0.9 ± 0.7	<0.001
LDL Cholesterol (nmol/L)	2.7 ± 0.8	2.9 ± 1.2	3.3 ± 0.5	<0.001
BMI (kg/m^2^)	22.7 ± 3.1	27.3 ± 3.6	29.4 ± 4.4	<0.001
Waist circumference (cm)	91 ± 8.9	97.3 ± 9.7	101 ± 13.2	<0.001
Central obesity (%)	63 (51.6)	189 (84.7)	230 (90.2)	<0.001
Dietary intake				
Total energy intake (kcal/day)	1901.4 ± 300.6	1995.3 ± 435.7	2184.9 ± 538.5	0.1296
Proteins (g/day)	89.9 ± 20.7	96.5 ± 27.4	104 ± 35.8	0.0372
Proteins (% total energy intake)	18.9 ± 4.2	19.3 ± 5.4	29.7 ± 3.9	0.0712
Carbohydrates (g/day)	271.7 ± 73.2	283.6 ± 83.6	280.3 ± 93.5	0.3972
Carbohydrates (% total energy intake)	57.1 ± 5.7	56.7 ± 6.2	51.3 ± 8.9	0.1287
Fats (g/day)	65.9 ± 37.2	78.3 ± 46.8	89.4 ± 38.9	0.0367
Fats (% total energy intake)	31.2 ± 7.3	35.2 ± 5.8	37.1 ± 3.9	0.0481
Nutrition knowledge				
Insufficient (%)	10 (8.2)	77 (34.5)	125 (49.1)	<0.001
Sufficient (%)	81 (66.4)	101 (45.3)	98 (38.4)	
High (%)	31 (25.4)	45 (20.2)	32 (12.5)	

HDL—High Density Lipoprotein, LDL—Low Density Lipoprotein, BMI—body mass index.

**Table 4:** Odds ratio (95% confidence interval) for dietary-lifestyle patterns by demographic factors, metabolic syndrome occurrence and nutrition knowledge.

Variables	Prudent-Active (Ref.: NotPrudent-NotWestern-LowActive)	Western-Sedentary (Ref.: NotPrudent-NotWestern-LowActive)	Western-Sedentary (Ref.: Prudent-Active)
Women (ref.: men)	1.37 * (1.21; 1.82)	0.83 ** (0.64; 0.96)	0.47 ** (0.31; 0.67)
Metabolic syndrome (ref. without MS)	0.27 * (0.21; 0.68)	1.27 * (0.82; 1.56)	2.46 *** (2.03; 2.77)
Sufficient nutrition knowledge (ref.: insufficient)	1.53 ** (1.12; 2.14)	0.76 ** (0.41; 0.92)	0.42 *** (0.21; 0.62)
High nutrition knowledge (ref.: insufficient)	2.23 ** (1.56; 3.13)	0.43 * (0.12; 0.87)	0.23 *** (0.14; 0.34)

* *p* < 0.05; ** *p* < 0.01; *** *p* < 0.001.

**Table 5:** Odds ratio (95% confidence interval) for dietary-lifestyle patterns by metabolic syndrome criteria.

Variables	Central Obesity (Ref.: Lack)	Hypertension (Ref.: Lack)	Hyper-triglicerydemia (Ref.: Normal)	Low HDL-Cholesterol (Ref.: Normal)	Hyperglycemia (Ref.: Normal)
Prudent-Active (ref.: NotPrudent-notWestern-lowActive)	0.57 ** (0.34; 0.88)	0.52 ** (0.23; 0.82)	0.59 *** (0.28; 0.95)	0.71 *** (0.36; 0.89)	0.62 ** (0.31; 0.97)
Western-Sedentary (ref.: NotPrudent-notWestern-lowActive)	1.08 (0.68; 1.78)	0.91 (0.21; 0.99)	0.98 (0.56; 1.35)	1.17 * (0.86; 1.42)	0.83 (0.67; 1.04)
Western-Sedentary (ref.: Prudent-Active)	2.09 ** (1.27; 3.25)	1.76 ** (1.23; 2.38)	2.16 *** (1.85; 2.58)	2.21 *** (2.03; 2.59)	1.34 ** (1.07; 1.61)

* *p* < 0.05; ** *p* < 0.01; *** *p* < 0.001.

## Data Availability

Publicly available datasets were not analyzed in this study.
